# From Carbene-Dithiolene Zwitterion Mediated B–H Bond Activation to BH_3_·SMe_2_-Assisted Boron–Boron Bond Formation

**DOI:** 10.1021/acs.organomet.3c00361

**Published:** 2023-10-11

**Authors:** Yuzhong Wang, Phuong M. Tran, Mitchell E. Lahm, Pingrong Wei, Earle R. Adams, Henry F. Schaefer, Gregory H. Robinson

**Affiliations:** Department of Chemistry and Center for Computational Chemistry, The University of Georgia, Athens, Georgia 30602-2556, United States

## Abstract

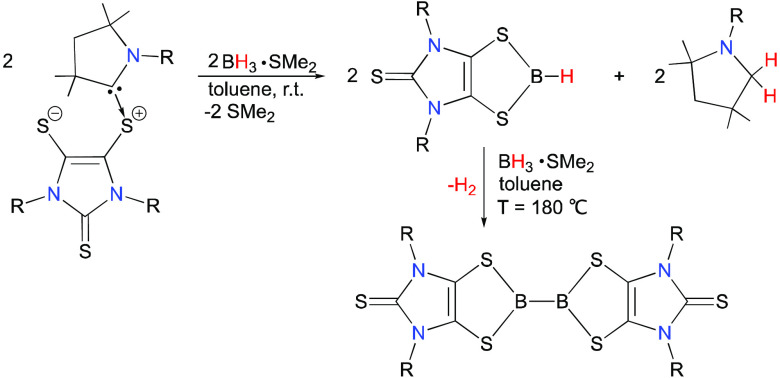

The 1:1 reaction of the carbene-stabilized dithiolene zwitterion **1** with BH_3_·SMe_2_ gave the dithiolene-based hydroborane **2** and the doubly hydrogen-capped CAAC species **3** via hydride-coupled reverse electron transfer processes. The mechanism of this transformation was probed computationally using density functional theory. The subsequent 2:1 reaction of **2** with **1** resulted in **4** and **3**, suggesting that **1** can mediate the B–H bond activation not only for BH_3_ but also for monohydroboranes. In the presence of BH_3_·SMe_2_, **2** was unexpectedly converted to the corresponding diborane(4) complex **5** through a dehydrocoupling reaction at an elevated temperature.

## Introduction

**Scheme 1:**
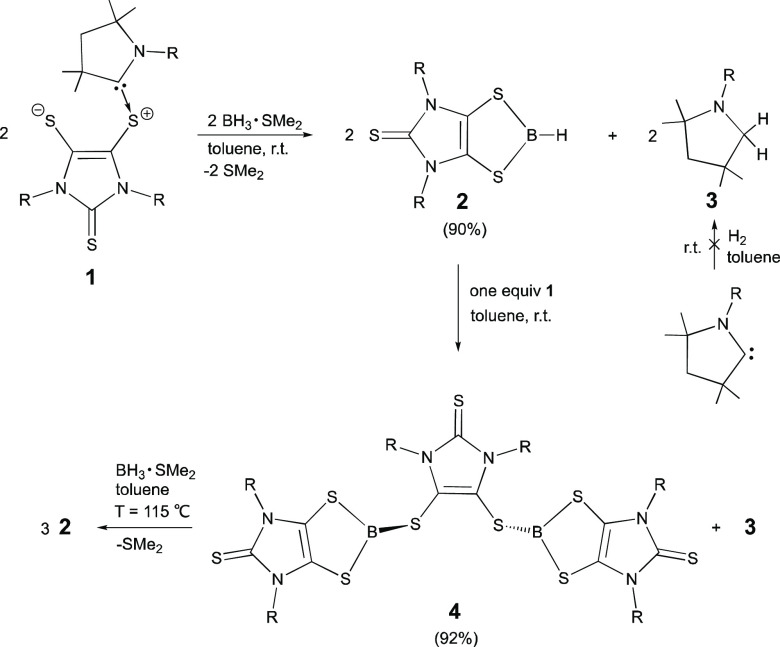
Synthesis of **2** and **4** (R = 2,6-Diisopropylphenyl)

The chemistry of boranes, compounds with boron–hydrogen bonds, has progressed significantly over the past century.^1−3^ Correspondingly, due to the pivotal role of boranes in a myriad of synthetic applications,^4^ coupled with the potential of ammonia–borane and related compounds as carbon-free energy sources,^5^ activation of the B–H bond steadfastly remains extremely relevant. Although higher order boranes are readily obtained from the pyrolysis of diborane (B_2_H_6_),^6^ the B–H bond is among the strongest two-electron σ-bonds on record (B–H bond dissociation energy (BDE): ca. 106 kcal mol^–1^).^6,7^ Traditionally, transition-metal species have played a dominant role in B–H bond activation, wherein the B–H bond is commonly activated through an oxidative addition mechanism. Indeed, oxidative addition of a borane B–H unit to a metal center has been reported as a key step in transition-metal-catalyzed hydroboration.^8,9^ Furthermore, metal–ligand cooperative B–H bond activation reactions are receiving increased attention.^10^ While main-group-species^11−18^-mediated B–H bond activation mainly involves 1,1-addition at a low-valent main-group species (such as carbenes,^11^ carbenoid species,^12^ and amidosilylenes^13^), frustrated Lewis pairs (FLPs)^14,15^ and boron-containing heterocycles^16,17^ have also been reported for this utility. In addition, boranes may demonstrate reduced B–H BDE values when being complexed by Lewis bases (such as NHCs).^19,20^ Consequently, NHC-complexed boranes have been employed as hydrogen atom donors via homolytic B–H cleavage.^20−22^ Notably, nitrogen-centered radical-mediated B–H activation of icosahedral carboranes via hydrogen atom transfer (HAT) has recently been reported.^23^ This laboratory recently reported a cyclic (alkyl)(amino)carbene (CAAC)-stabilized dithiolene zwitterion (**1**, in Scheme 1),^24^ a new type of metal-free bifunctional molecular system which has been shown to activate ammonia via both single-electron-transfer (SET) and HAT processes.^25^ Therein the 4π-electron neutral dithiolene moiety serves as an electron reservoir, while the in situ released CAAC ligand acts as a nucleophile. Herein, we report the CAAC-dithiolene zwitterion (**1**) mediated double and triple B–H bond activation of BH_3_ via hydride-coupled reverse electron transfer (HCRET) processes. Interestingly, the ^Me^CAAC^26−28^ ligand in **1** may serve as a two-hydrogen-atom acceptor in these transformations. Although the 1,2-hydride migration from a boron center to the adjacent carbene carbon of the CAAC ligand has been documented,^29,30^ observation of CAAC as a double-hydrogen-atom acceptor via HCRET has not been reported yet.

## Results and Discussion

The room-temperature reaction of **1** with BH_3_·SMe_2_ (in a 1:1 ratio) in toluene rapidly gave the dithiolene-based hydroborane **2** as an off-white powder in 90% yield (Scheme 1)^31^ and the byproduct **3**.^32^ The ^1^H NMR spectroscopic study shows that **2** and **3** are formed in a ca. 1:1 ratio. The doublet ^11^B NMR resonance of the B(H) unit for **2** at 51.5 ppm (^1^*J*_BH_ = 152 Hz, in toluene-*d*_8_, at 100 °C) compares to that (61.3 ppm (^1^*J*_BH_ = 140 Hz)) for (CH_2_S)_2_BH (1,3,2-dithiaborolane).^33^ In addition, the B–H stretching absorption (2469 cm^–1^) in the infrared (IR) spectrum (Figure S14) of **2** (in KBr) is comparable with that (2435 cm^–1^) of (CH_2_S)_2_BH.^34^ Further reaction of **2** with **1** (in a 2:1 ratio) in toluene at room temperature gave **4** as an off-white powder (in 92% yield) and byproduct **3** (Scheme 1).^31^ The ^11^B NMR resonance (in C_6_D_6_) of **4** displays a broad singlet at 59.3 ppm (*w*_1/2_ = 991 Hz). Compound **4** may also be directly prepared by the reaction of **1** with BH_3_·SMe_2_ in a 3:2 ratio. Compound **4** was quantitatively converted to **2** via a 1:1 reaction with BH_3_·SMe_2_ at an elevated temperature (*T* = 115 °C) (Scheme 1). Isolation of the doubly-hydrogen-capped CAAC species **3** as a byproduct in the **1**-to-**2** and **2**-to-**4** conversions suggests that two hydrogen atoms are ultimately transferred to the CAAC ligand.

**Figure 1:**
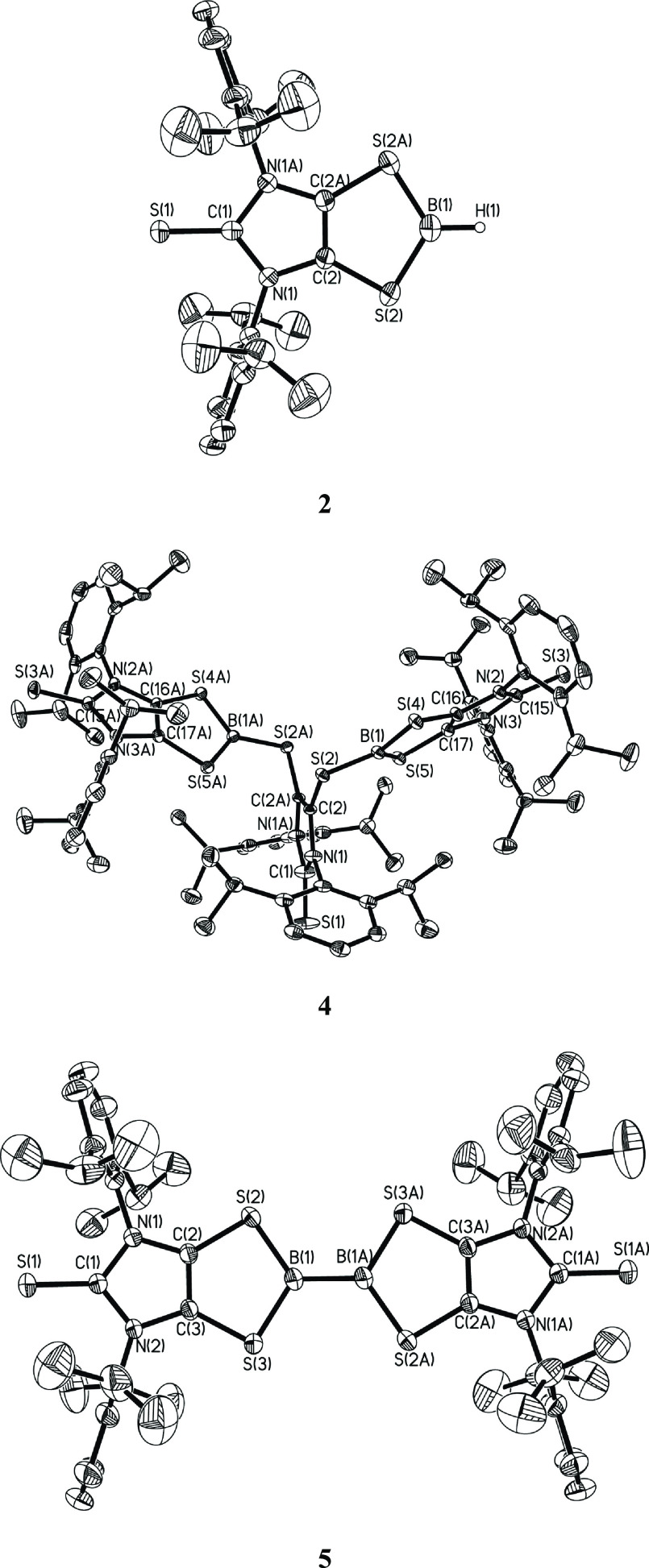
Molecular structures of **2**, **4**, and **5**. Thermal ellipsoids represent 30% probability. Hydrogen atoms on carbon have been omitted for clarity. Selected bond distances (Å) and angles (deg) for **2**: C(2)–C(2A) 1.331(5); C(2)–S(2) 1.724(2); S(2)–B(1) 1.792(3); C(2A)–C(2)–S(2) 119.74(8); S(2A)–B(1)–S(2) 116.1(3). Selected bond distances (Å) and angles (deg) for **4**: C(2)–C(2A) 1.342(8); C(2)–S(2) 1.759(4); S(2)–B(1) 1.815(5); C(2A)–C(2)–S(2) 130.58(14); C(2)–S(2)–B(1) 100.4(2). Selected bond distances (Å) and angles (deg) for **5**: B(1)–B(1A) 1.645(6); S(2)–B(1) 1.796(3); C(2)–S(2) 1.715(3); S(2)–B(1)–S(3) 113.88(16); S(2)–B(1)–B(1A) 123.6(3); S(3)–B(1)–B(1A) 122.5(3).

Compounds **2** and **4** were further characterized by single-crystal X-ray diffraction analysis.^31^ The hydrogen atom bound to the boron atom in **2** (Figure 1) was located from difference Fourier maps. The three-coordinate boron atom in **2** adopts a trigonal-planar geometry. The experimental (1.792(3) Å) and theoretical (1.808 Å) values of the S–B bond distance of **2** are considerably shorter than those in four-coordinate boron-based dithiolene complexes (1.927(4)–2.031(2) Å)^35^ due to the S–B π interaction (The S–B Wiberg bond index (WBI) of **2** is 1.29). In the solid state, compound **4** exists as a pair of enantiomers with identical bonding parameters (for clarity, only one of the enantiomers is shown in Figure 1). The X-ray structural analysis of **4** shows that the dithiolene unit serves as a bridging ligand to bind two boron dithiolene species via two S–B single bonds (1.815(5) Å) on opposite sides of the dithiolene (C_2_S_2_) plane (in *C*_2_ symmetry), confirming exhaustive B–H bond activation of BH_3_.

The room-temperature NMR-tube reaction of **1** with BH_3_·SMe_2_ (in a 1:1 ratio) in C_6_D_6_ did not exhibit the singlet ^1^H NMR resonance of H_2_(g) at 4.47 ppm (Figure S13)^31^ and thus does not support the evolution of dihydrogen gas in the formation of **2**. Although CAAC-mediated dihydrogen splitting has been observed at 35 °C,^36^ the free ^Me^CAAC ligand does not react with H_2_(g) in toluene at room temperature (Scheme 1).

**Figure 2:**
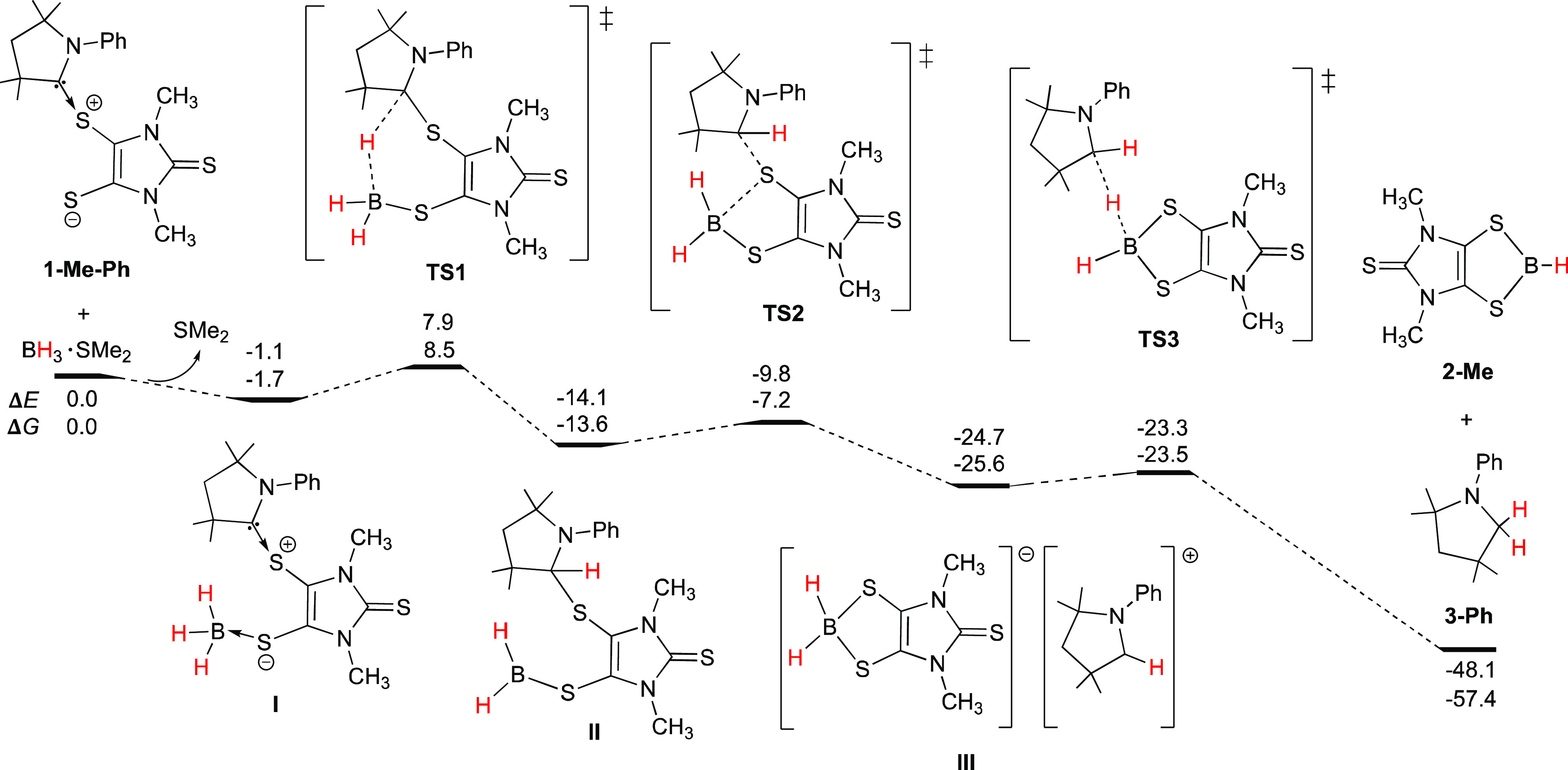
Computed relative energies (Δ*E*, in kcal mol^–1^) and free energies (Δ*G*, in kcal mol^–1^) for the reaction of the simplified **1-Me-Ph** model with BH_3_·SMe_2_ at the B3LYP/6-311G** (SMD, toluene) level.

Toward a plausible mechanism, the reaction of the simplified **1-Me-Ph** model with BH_3_·SMe_2_ was probed using density functional theory (DFT) at the B3LYP/6-311G** (SMD, toluene) level (Figure 2).^31^

To this end, Lewis adduct **I** is generated via the weakly exergonic reaction of **1-Me-Ph** with BH_3_·SMe_2_ (Δ*G* = −1.7 kcal mol^–1^). The formation of **I** involves the coordination of the terminal sulfur atom of the C_2_S_2_ unit in **1-Me-Ph** to one BH_3_ molecule, while releasing dimethyl sulfide (SMe_2_) as a byproduct. The adduct **I** may be readily converted to intermediate **II** via intramolecular B-to-C_CAAC_ hydrogen migration (**TS1** (transition state) energy barrier 10.2 kcal mol^–1^). The **I**-to-**II** conversion is exergonic by 11.9 kcal mol^–1^. Compound **II** could be subsequently converted to intermediate **III** (with an energy of −25.6 kcal mol^–1^) via an intramolecular cyclization transition state (i.e., **TS2** with an energy barrier of 6.4 kcal mol^–1^). In the **II**-to-**III** conversion, accompanying the formation of the five-membered C_2_S_2_B ring (as observed in **III**), the S–C_CAAC_ bond is gradually elongated (*d*_S···C(CAAC)_ = 1.948 Å for **TS2** vs *d*_S–C(CAAC)_ = 1.901 Å for **II**) and finally heterolytically cleaved, which is supported by a natural bond orbital (NBO) analysis of **III**. NBO analysis shows that the CAAC-H and dithiolene-BH_2_ moieties in **III** have a positive charge of +0.88 and a negative charge of −0.88, respectively. Thus, the formation of **III** involves CAAC-H-to-dithiolene-BH_2_ one-electron transfer. Subsequently, the cationic CAAC-H unit of **III** may readily take one hydride group from the boron atom (via the transition state **TS3** with an energy barrier of 2.1 kcal mol^–1^), rendering the final products **2-Me** and **3-Ph**. This conversion is also thermodynamically favored (Δ*G* = −31.8 kcal mol^–1^). The DFT computations indicate that the net transfer of two hydrogen atoms from borane to the CAAC ligand (as observed in the formation of **3**) may go through a hydride-coupled reverse electron transfer (HCRET) process in which the hydride migrates from the boron atom to the CAAC ligand, while one electron is transferred from the CAAC unit back to the boron-dithiolene fragment. It is noteworthy that both the electrophilic property of the CAAC ligand^37^ and the redox-active character of the dithiolene ligand^38^ play pivotal roles in the hydride-coupled reverse electron transfer reaction. For example, in the **1**-to-**2** conversion the dithiolene fragment is reduced from a neutral dithiolene zwitterion (as shown in **1**) to a dithiolate (as shown in **2**). The **2**-to-**4** conversion shows that **1** can activate the boron–hydrogen bond not only for BH_3_ but also for monohydroboranes via similar HCRET processes. In contrast to the high energy barrier for the homolytic B–H cleavage in HAT, the HCRET process provides an energetically effective route for net “hydrogen atom” transfer.

**Scheme 2:**
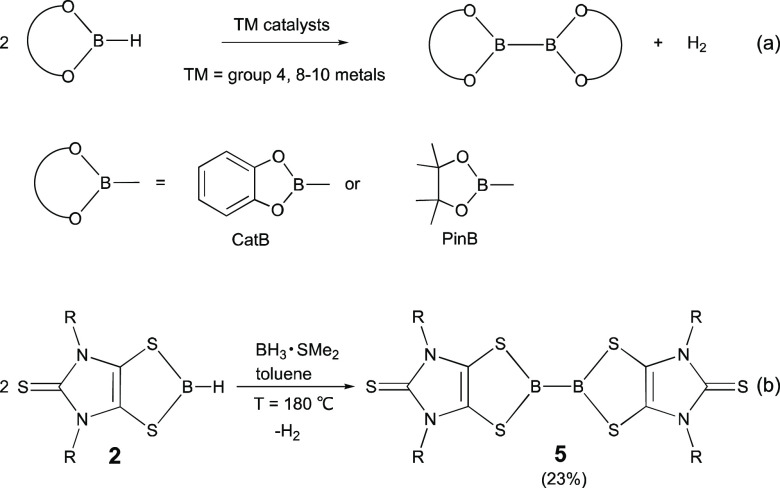
Electron-Precise Boron–Boron Bond Formation: (a) Transition-Metal-Catalyzed Borane Dehydrocoupling Reactions; (b) Borane-Mediated Dehydrocoupling of **2** (R = 2,6-Diisopropylphenyl)

Diboranes(4) have demonstrated extensive utility in organic synthesis.^39^ While haloborane reduction remains the principal synthetic means for electron-precise boron–boron bond formation, the borane dehydrocoupling method has received increasing attention.^40^ For example, catecholborane (CatBH) and pinacolborane (PinBH) have been converted to the corresponding diboranes(4) via transition-metal-catalyzed dehydrocoupling reactions (Scheme 2a).^41,42^ Metal-free formation of boron–boron bonds via borane dehydrocoupling reactions is rare.^43,44^ The calculated B–H bond dissociation energy in **2** (112.2 kcal mol^–1^)^31^ compares well to that of catecholborane (CatBH) (111.3 kcal mol^–1^).^7^ This study demonstrated that, in the presence of a small amount of BH_3_·SMe_2_, pyrolysis of **2** in toluene at 180 °C in a sealed Schlenk tube for 30 min gave an orange-red mixture, consisting of the dithiolene-based diborane(4) **5**, the unreacted **2**, and other unidentified byproducts (Scheme 2b). **5** was isolated as orange-red crystals (in 23% yield) from the concentrated parent solution. It is noteworthy that, in the absence of BH_3_·SMe_2_, the **2**-to-**5** conversion in toluene did not occur even at a high temperature. The NMR-tube reactions indicate that the reaction stoichiometry influences the **2**-to-**5** conversion. Reaction of **2** with BH_3_·SMe_2_ (in a molar ratio of 6:1) gave a relatively high **2**-to-**5** conversion. The resulting reaction mixture contains both **2** and **5** in a 1:1.2 molar ratio. Notably, BH_3_·SMe_2_ is expected to decompose in toluene at 180 °C (BDE of the B–S bond in BH_3_·SMe_2_: 21.2 kcal mol^–1^).^31^ The released BH_3_ species may be further transformed into a complicated mixture of higher order boranes, even polymeric BH_*x*_ materials.^6^ Due to the complexity of this pyrolysis reaction, mechanistic aspects of the **2**-to-**5** conversion remain unclear. H_2_ gas is expected to be formed as a byproduct in the **2**-to-**5** conversion (Scheme 2). The ^1^H NMR signal of H_2_ was indeed observed as a singlet at 4.51 ppm (in toluene-*d*_8_) in the high-temperature NMR-tube reaction of **2** with BH_3_·SMe_2_. However, the pyrolysis of BH_3_·SMe_2_ itself can also produce H_2_.

**Figure 3:**
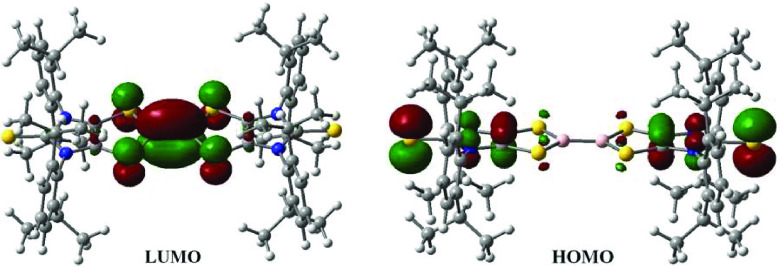
Frontier molecular orbitals of **5**.

The UV–vis absorption spectrum of **5** (orange-red) in toluene shows one strong broad absorption at 478 nm (Figure S1). It compares to the TD-DFT calculated value of 536 nm (B3LYP/6-311G**, SMD, toluene), corresponding to the excitation from HOMO to LUMO. While the HOMO is dithiolene ligand based, the LUMO mainly involves B–B π-bonding and S–B π-antibonding character (Figure 3). The X-ray structural analysis of **5** (Figure 1) shows that all atoms are essentially coplanar, except for those of the four flanking Dipp substituents (Dipp = 2,6-diisopropylphenyl). Like **2** and **4**, each three-coordinate boron atom in **5** adopts a trigonal-planar geometry. The B–B (1.645(6) Å) and B–S (1.798 Å, av) bonds in **5** are comparable with the calculated values of **5** (*d*_B–B_ = 1.665 Å; *d*_B–S_ = 1.825 Å) and those of bis(dithiocatecholato)diborane, B_2_(1,2-S_2_C_6_H_4_)_2_ (**6**) (*d*_B–B_ = 1.672 Å, av; *d*_B–S_ = 1.794 Å, av).^45^ The ^11^B NMR spectrum of **5** (in toluene-*d*_8_, 100 °C) exhibits a singlet resonance at 54.6 ppm (*w*_1/2_ = 471 Hz), which is comparable with that of **6** (57.9 ppm).^46^

## Conclusion

The CAAC-stabilized neutral dithiolene **1** may react with BH_3_·SMe_2_, giving the dithiolene-based boranes (**2** and **4**) and doubly hydrogen-capped CAAC species **3** (as a byproduct) via a hydride-coupled reverse electron transfer (HCRET). The borane-mediated boron–boron bond formation in **5** via the high-temperature dehydrocoupling of dithiolene-based hydroborane (**2**) represents yet another surprise. The unique synergic interaction between the noninnocent dithiolene unit and the electrophilic CAAC ligand in **1** is expected to exhibit other unusual applications for small-molecule activation.

## Supporting Information Available

Syntheses, computations, and X-ray crystal determination (PDF)
Cartesian coordinates for the calculated structures (XYZ)

## Supplementary Material

om3c00361_si_001.pdf

om3c00361_si_002.xyz
