# Supplementary material for: From Carbene-Dithiolene Zwitterion Mediated B–H Bond Activation to BH3·SMe2-Assisted Boron–Boron Bond Formation
Source: Organometallics. 2023 Oct 11;42(23):3328–33. doi: 10.1021/acs.organomet.3c00361 (PMC10716900; doi:10.1021/acs.organomet.3c00361)
Supplement: Supplementary file 1 — om3c00361_si_001.pdf [file om3c00361_si_001.pdf]

Supplementary Information

From Carbene-Dithiolene Zwitterion-Mediated B–H Bond Activation to $BH_3{\cdot}SMe_2$-Assisted Boron–Boron Bond Formation

Yuzhong Wang, Phuong M. Tran, Mitchell E. Lahm, Pingrong Wei, Earle R. Adams, Henry F. Schaefer III, and Gregory H. Robinson*

Department of Chemistry, Center for Computational Chemistry, and Complex Carbohydrate Research Center, The University of Georgia, Athens, Georgia 30602-2556 (USA)

*To whom correspondence should be addressed.
Email: robinson@uga.edu

Table of Contents **Page**

Synthesis and spectral data S2-S17

Computational data S18

X-Ray Crystallography Data S19-S30

References: S30

SUPPORTING INFORMATION of SYNTHESES

Materials and Methods

General.

All reactions were performed under purified argon using Schlenk techniques and an inert atmosphere drybox (M-Braun LabMaster 130). Chemicals were purchased from commercial sources and used as received. Solvents were dried and distilled under argon from Na/benzophenone prior to use. ^{1}H, ^{13}C, and ^{11}B NMR spectra were recorded on a Bruker Avance III HD 400 MHz spectrometer. The ^{11}B NMR spectra were referenced to $BF_3 \cdot OEt_2$ as an external standard. Infrared spectrum was recorded using a Shimadzu IRPrestige-21 FTIR spectrophotometer. UV-visible absorption spectrum was recorded under argon gas protection in 1 cm cuvettes using an Agilent Cary 60 UV-Vis spectrophotometer. X-ray intensity data for **2·(toluene)** (CCDC# 2269318) and **5·(toluene)₂** (CCDC# 2269321) were collected at 299K and **4** (CCDC# 2269320) was collected at 135K on a Bruker D8 Quest PHOTON 100 CMOS X-ray diffractometer system with Incoatec Microfocus Source (IμS) monochromated Mo Kα radiation (λ = 0.71073 Å, sealed tube) using phi and omega-scan technique.

Compounds **2** and **3**: The 5mL toluene solution of $BH_3 \cdot SMe_2$ (0.050 g, 0.69 mmol) was added to a Schlenk tube containing **1** (0.500 g, 0.65 mmol) in 15 mL of toluene at ambient temperature. The slurry was stirred for 24 h, after which volatiles were removed in vacuo. The residue was rinsed with 30 mL of hexane and then filtered, giving **2** as a white residue (0.289 g, 90% yield). X-ray quality crystals of **2** were obtained by concentrating the parent toluene solution. After hexane was removed from the filtrate in vacuo, an oily residue was obtained, which was then sublimed to give colorless X-ray quality crystals of **3** (0.11 g, 59% yield). Characterization of **2**: Mp: gradually decomposed (> 191 °C) and gradually melt (> 218 °C). ^{1}H NMR (400.22 MHz, toluene-d_8, 25 ºC): δ 1.15 [d, 12H, CH(C*H*$_3$)$_2$], 1.39 [d, 12H, CH(C*H*$_3$)$_2$], 2.90 [m, 4H, C*H*(CH$_3$)$_2$], 7.14 (d, 4H, Ar-*H*), 7.24 (t, 2H, Ar-*H*). ^{13}C{^{1}H} NMR (100.65 MHz, toluene-d_8, 100 ºC): δ 24.1, 24.2 [CH(*C*H$_3$)$_2$], 29.8 [*C*H(CH$_3$)$_2$], 124.9, 131.0, 133.3, 147.7, 173.7 (Ar-*C* and imidazole-*C*). ^{11}B NMR (128.41 MHz, toluene-d_8, 100 ºC): δ 51.5 ppm (d, $^{1}J_{BH}$ = 152 Hz). ^{11}B{^{1}H} NMR (128.41 MHz, toluene-d_8, 100 ºC): δ 51.5 ppm ($w_{1/2}$ = 187 Hz). IR ν(KBr): 2469 cm^{-1} (w) (B–H stretching). Crystal data for **2·(toluene)**: $C_{34}H_{43}BN_2S_3$, fw = 586.69, monoclinic, *P*2/c (No. 13), a = 10.7109(8) Å, b = 10.5096(8) Å, c = 16.2052(12) Å, β = 107.465(2)°, V = 1740.1(2) Å^3, Z = 2, R1 = 0.0676 for 2629 data ($I > 2\sigma(I)$), wR$_2$ = 0.1973 (all data). Characterization of **3**: Mp: 56–57 °C. ^{13}C{^{1}H} NMR (100.65 MHz, C_6D_6, 25 °C): δ 23.6, 27.2 [CH(*C*H$_3$)$_2$], 29.0 [*C*H(CH$_3$)$_2$], 29.4 [NC(*C*H$_3$)$_2$], 30.6 [CH$_2$C(*C*H$_3$)$_2$CH$_2$], 37.6 [CH$_2$*C*(CH$_3$)$_2$CH$_2$], 57.2 (C*C*H$_2$C), 63.7 [N*C*(CH$_3$)$_2$], 68.4 (N*C*H$_2$), 124.7 (C_{meta}, Ar), 127.5 (C_{para}, Ar), 139.4 (C_{ipso}, Ar), 152.7 (C_{ortho}, Ar). The ^{1}H NMR spectroscopic and X-ray data of **3** are consistent with the reported values of **3**.[1]

Compound **4**: 10 mL of toluene was added to a Schlenk tube containing **1** (0.155 g, 0.202 mmol) and **2** (0.200 g, 0.404 mmol). After the mixture were stirred at room temperature overnight, the volatiles were removed in vacuo. The residue was rinsed with 20 mL of hexane and subsequently dried in vacuo, giving **4** as an off-white powder (0.272 g, 92% yield). X-ray quality yellowish single crystals of **4** were obtained by concentrating the parent solution at room temperature. Mp: gradually decomposed (> 262 °C). ^{1}H NMR (400.22 MHz, toluene-d_8, 25 °C): δ 0.60 [d, 6H, CH(C*H*$_3$)$_2$], 1.01 [d, 6H, CH(C*H*$_3$)$_2$], 1.10 [broad s, 12H, CH(C*H*$_3$)$_2$], 1.17 [d, 12H, CH(C*H*$_3$)$_2$], 1.29 [d, 6H, CH(C*H*$_3$)$_2$], 1.38 [dd, 24H, CH(C*H*$_3$)$_2$], 1.44 [d, 6H, CH(C*H*$_3$)$_2$], 2.59–2.94 [m, 12H,

$CH(CH_3)_2$], 7.05–7.26 (m, 18H, Ar-*H*). $^{13}C\{^1H\}$ NMR (100.65 MHz, toluene-d$_8$, 25 °C): δ 23.4, 23.7, 24.0, 24.1, 24.2, 24.7, 25.0 [CH(CH_3)$_2$], 29.3, 29.6, 29.8 [$CH(CH_3)_2$], 123.1, 123.7, 124.6, 124.8, 130.8, 130.9, 131.9, 132.3, 147.1, 147.3, 147.4, 148.0, 171.4, 171.6 (imidazole-*C* and Ar-*C*). $^{11}B\{^1H\}$ NMR (128.41 MHz, C_6D_6, 25 ºC): δ 59.3 ppm ($w_{1/2}$ = 991 Hz). Crystal data for **4**: $C_{81}H_{102}B_2N_6S_9$, fw = 1469.84, monoclinic, *C*2/c (No. 15), a = 12.260(2) Å, b = 26.520(4) Å, c = 34.227(5) Å, β = 96.180(5)°, V = 11063(3) Å^3, Z = 4, R1 = 0.0880 for 8559 data ($I > 2\sigma(I)$), wR_2 = 0.2469 (all data).

Compound **5**: $BH_3 \cdot SMe_2$ (0.013 g, 0.17 mmol) in 4 mL of toluene was added to a Schlenk tube containing **2** (0.500 g, 1.01 mmol) in 4 mL of toluene. The Schlenk tube (with a closed side arm) was then heated at 180 ºC in an oil bath for 30 min. After keeping the Schlenk tube be stationary at room temperature overnight, the orange-red solution was filtered into a new Schlenk tube and then concentrated to 3 mL in vacuo. X-ray quality orange-red crystals of **5·(toluene)$_2$** (0.133 g, 23% yield) were obtained after storing the Schlenk tube at room temperature over one week. Mp: gradually decomposes > 217 °C and melts > 265 °C. UV-vis (λ/nm): 478. ^1H NMR (400.22 MHz, toluene-d$_8$, 25 ºC): δ 1.10 [d, 24H, CH(CH_3)$_2$], 1.37 [d, 24H, CH(CH_3)$_2$], 2.88 [m, 8H, $CH(CH_3)_2$], 7.13 (d, 8H, Ar-*H*), 7.25 (t, 4H, Ar-*H*). $^{13}C\{^1H\}$ NMR (100.65 MHz, toluene-d$_8$, 100 °C): δ 24.1, 24.2 [CH(CH_3)$_2$], 29.9 [$CH(CH_3)_2$], 124.7, 131.1, 133.2, 147.7, 174.1 (imidazole-*C* and Ar-*C*). $^{11}B\{^1H\}$ NMR (128.41 MHz, toluene-d$_8$, 100 ºC): δ 54.6 ppm ($w_{1/2}$ = 471 Hz). Crystal data for **5·(toluene)$_2$**: $C_{68}H_{84}B_2N_4S_6$, fw = 1171.36, triclinic, *P*-1 (No. 2), a = 9.0772(15) Å, b = 13.779(2) Å, c = 14.937(3) Å, α = 112.525(4)°, β = 96.635(5)°, γ = 92.610(4)°, V = 1705.9(5) Å^3, Z = 1, R1 = 0.0700 for 4961 data ($I > 2\sigma(I)$), wR_2 = 0.2090 (all data).

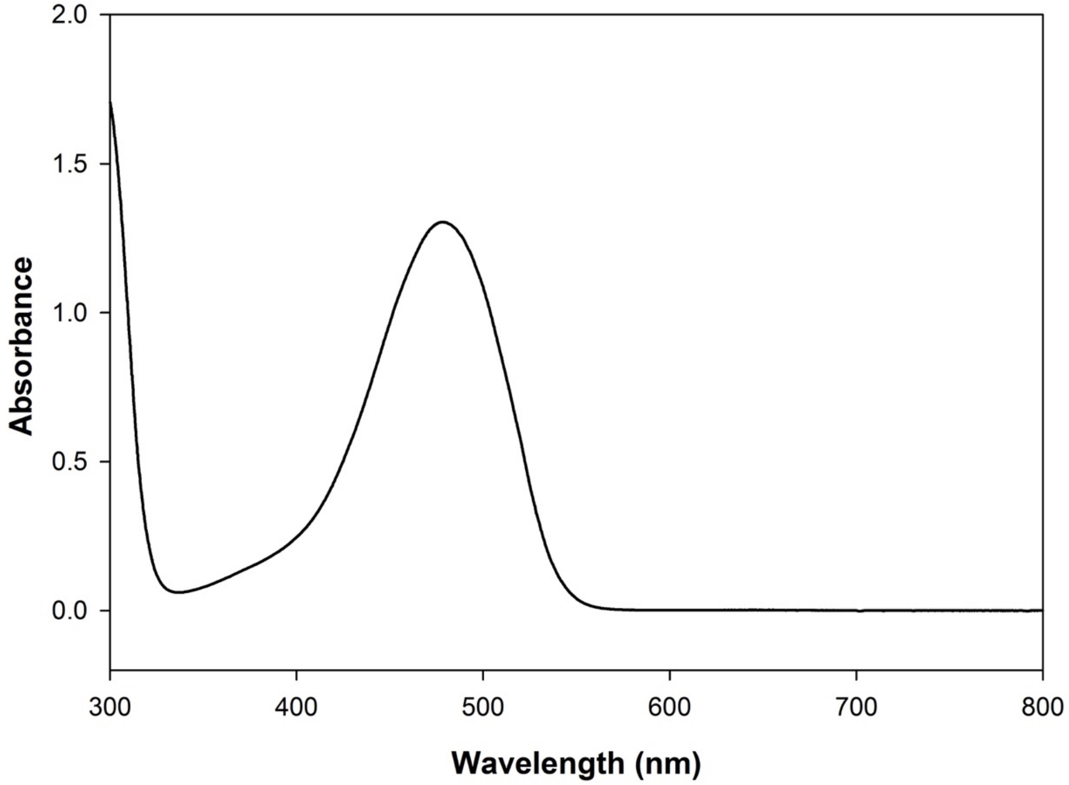

Fig. S1. UV-vis spectrum of **5** in toluene.

Compound **2**

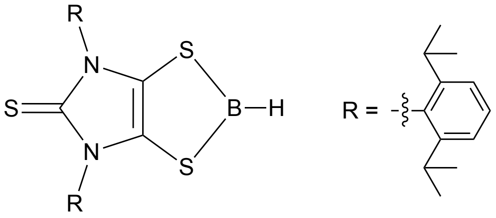

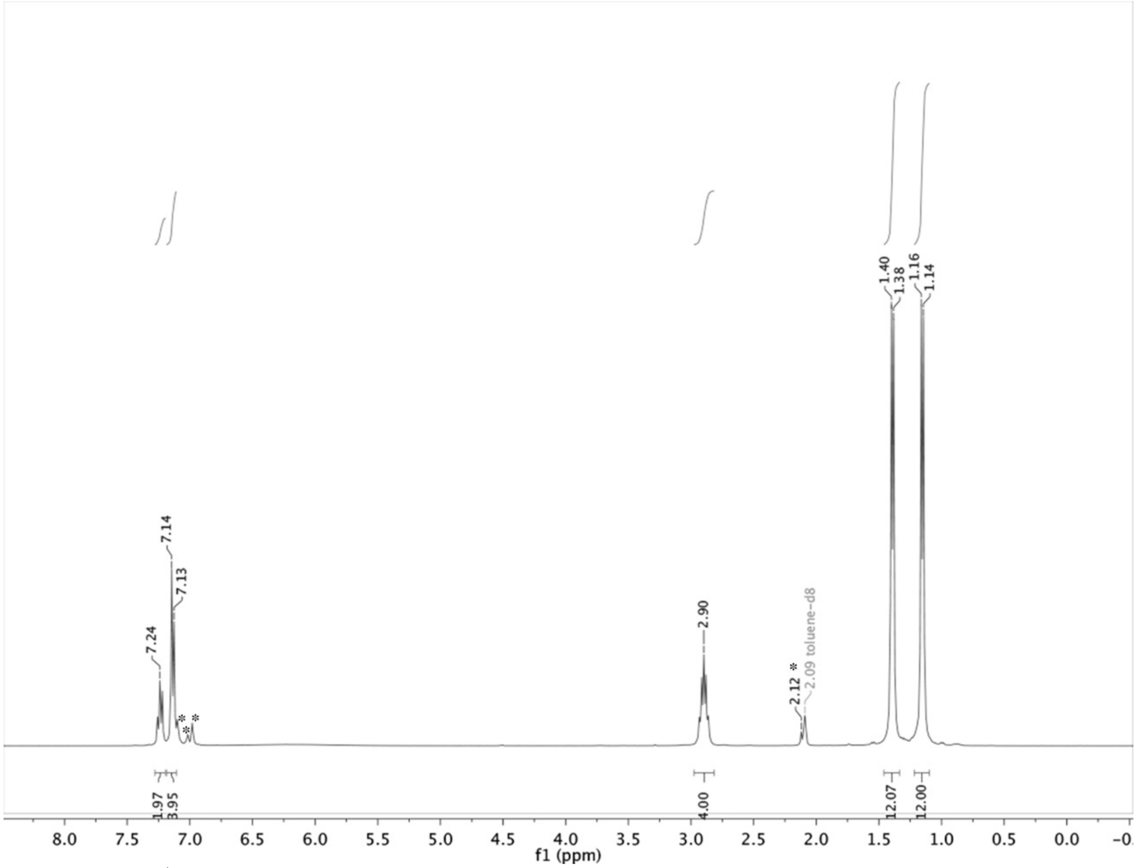

Fig. S2. ^{1}H NMR spectrum of **2** in toluene-d_8 at 25 ºC (*: toluene).

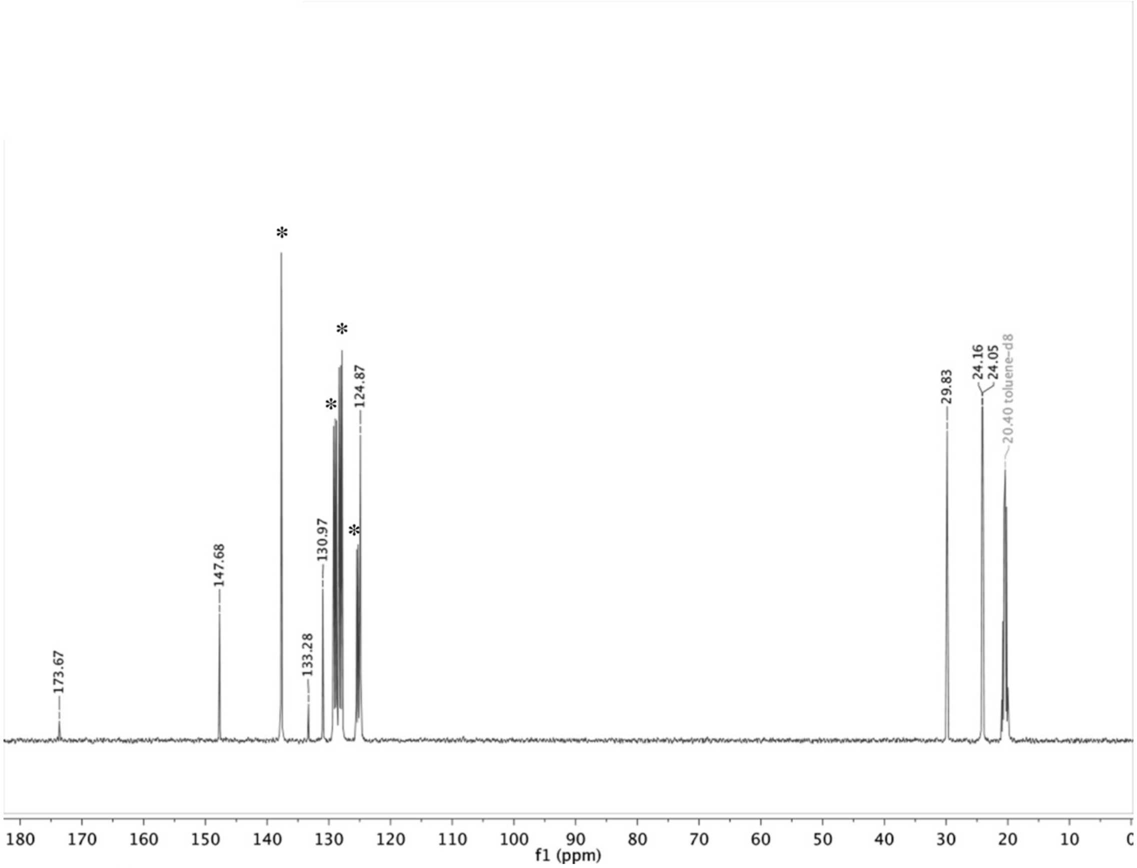

Fig. S3. ^{13}C NMR spectrum of **2** in toluene-d_8 at 100 ºC (*: toluene).

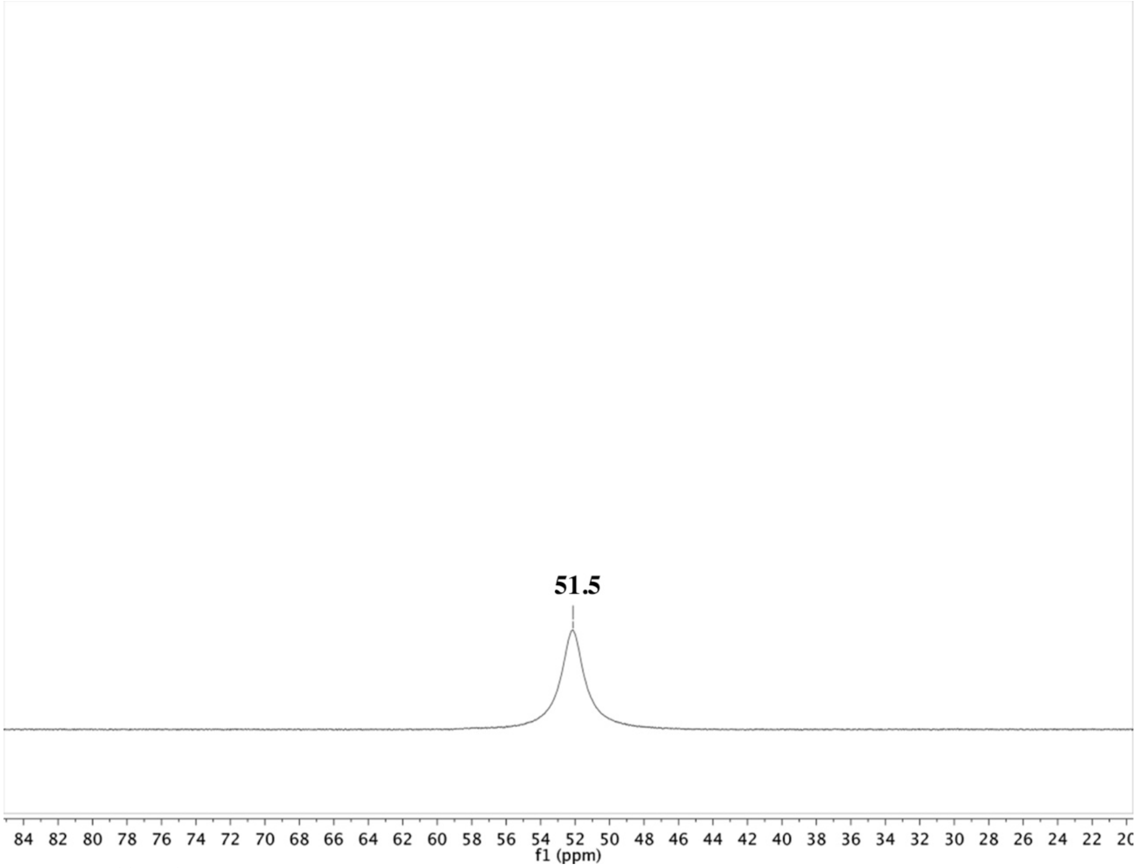

Fig. S4. $^{11}B\{^{1}H\}$ NMR spectrum of **2** in toluene-d_8 at 100 ºC.

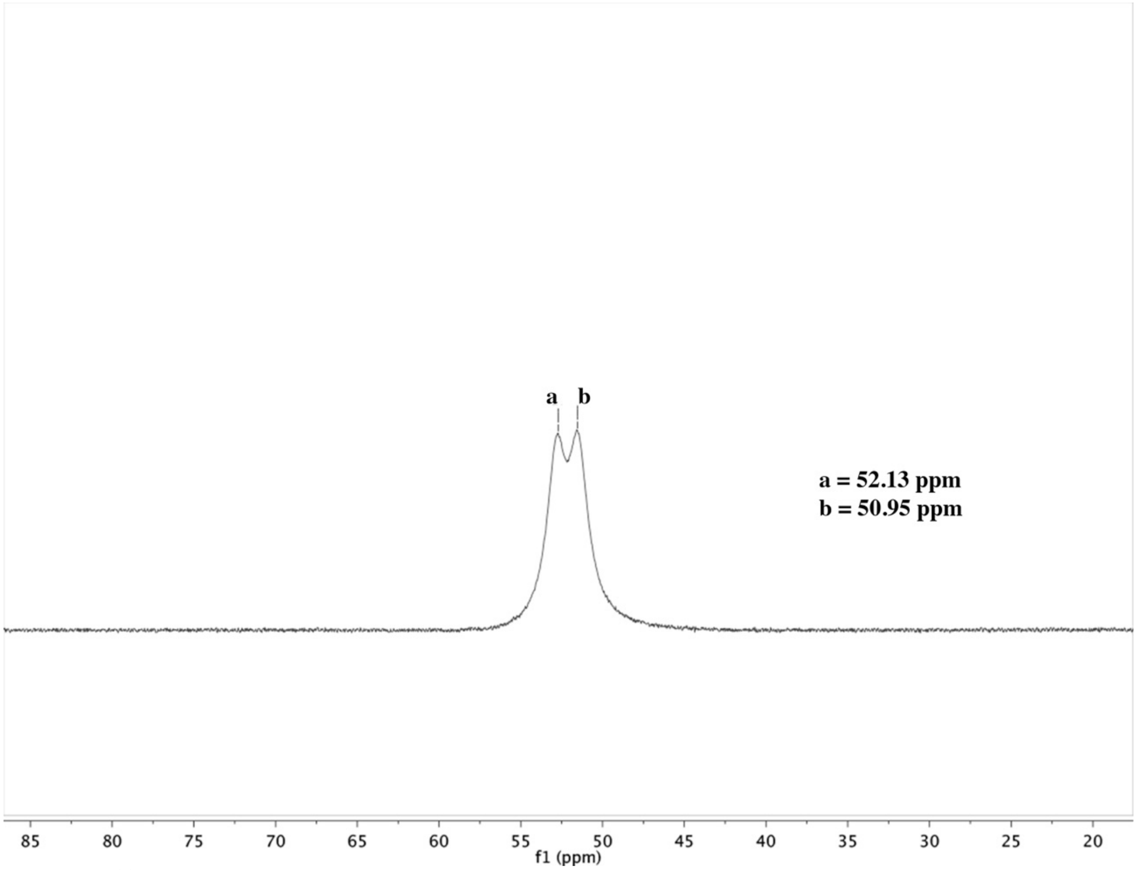

Fig. S5. ^{11}B NMR spectrum of **2** in toluene-d_8 at 100 ºC.

Compound **3**

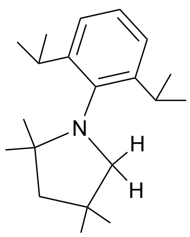

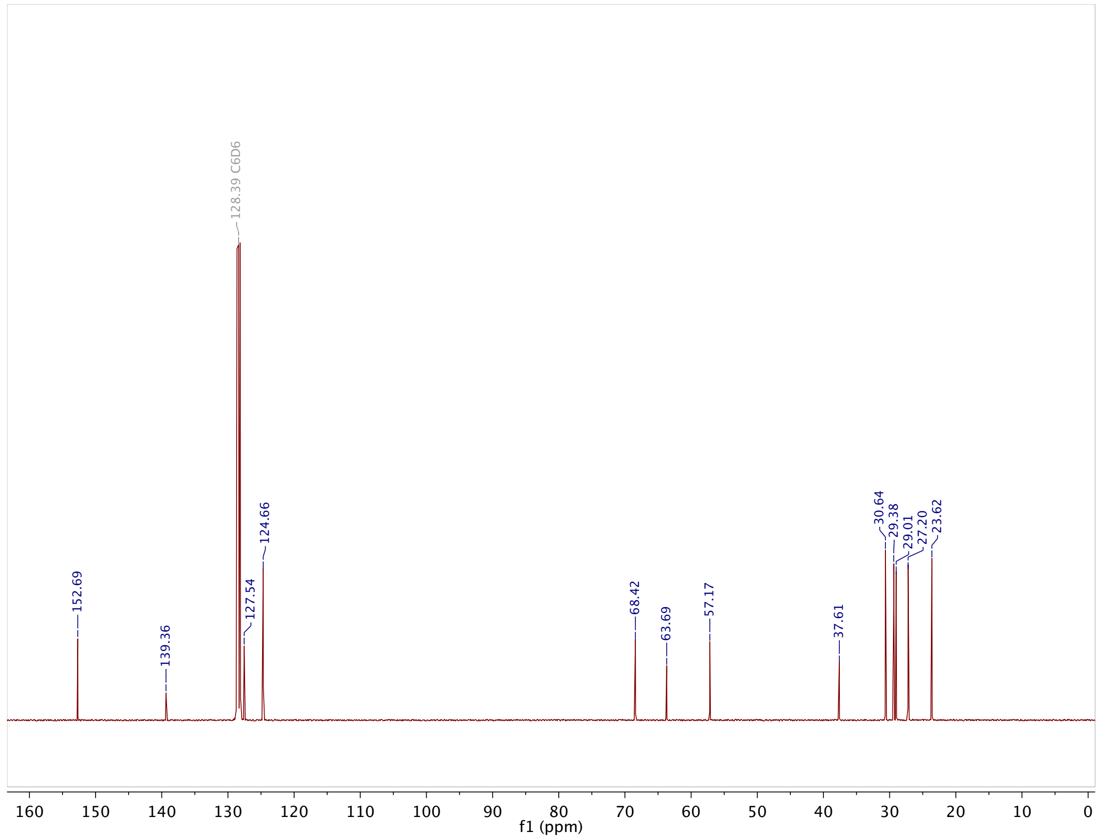

Fig. S6. ^{13}C NMR spectrum of **3** in C_6D_6 at 25 ºC.

Compound **4**

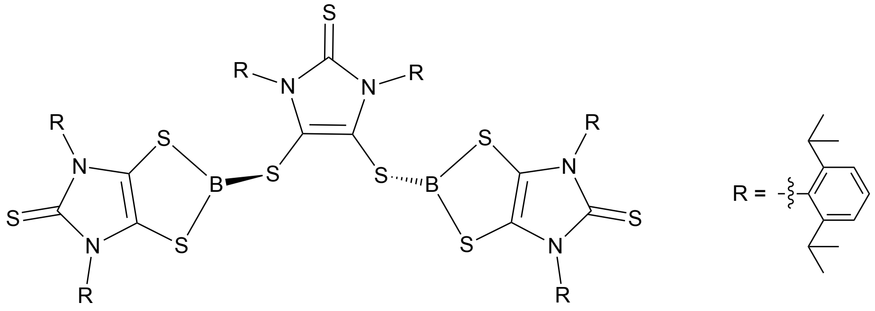

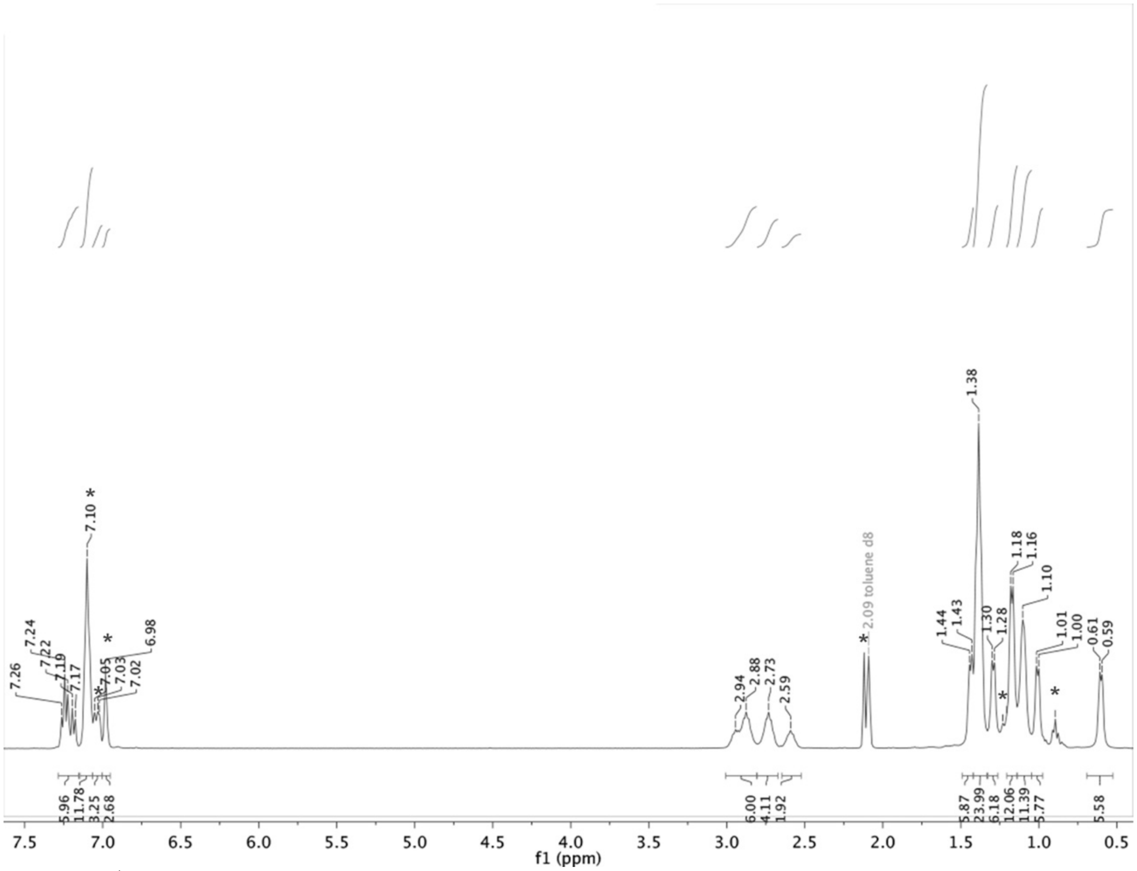

Fig. S7. ^{1}H NMR spectrum of **4** in toluene-d_8 at 25 ºC (*: toluene and hexane).

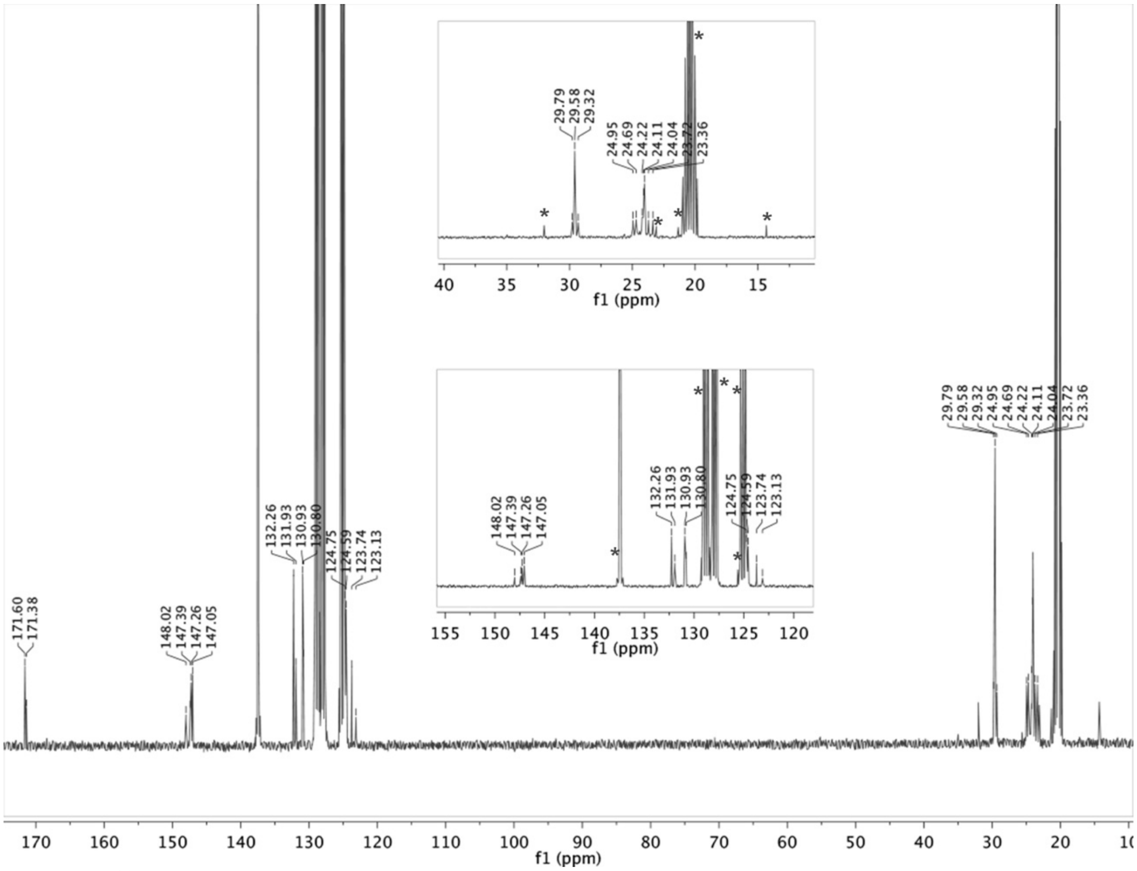

Fig. S8. ^{13}C NMR spectrum of **4** in toluene-d_8 at 25 ºC (*: toluene and hexane).

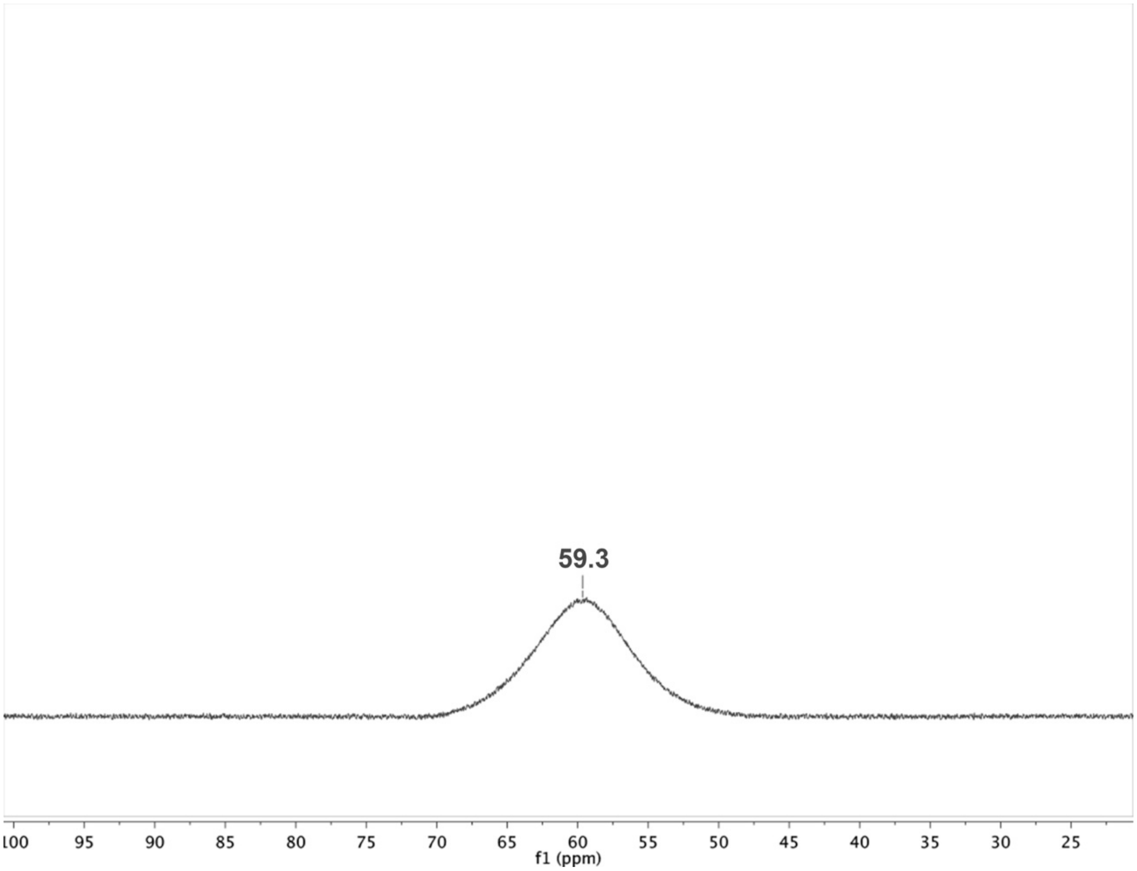

Fig. S9. $^{11}B\{^1H\}$ NMR spectrum of **4** in C_6D_6 at 25 ºC.

Compound **5**

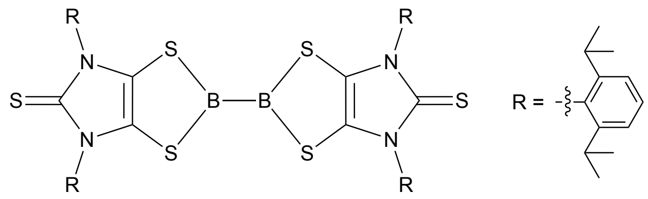

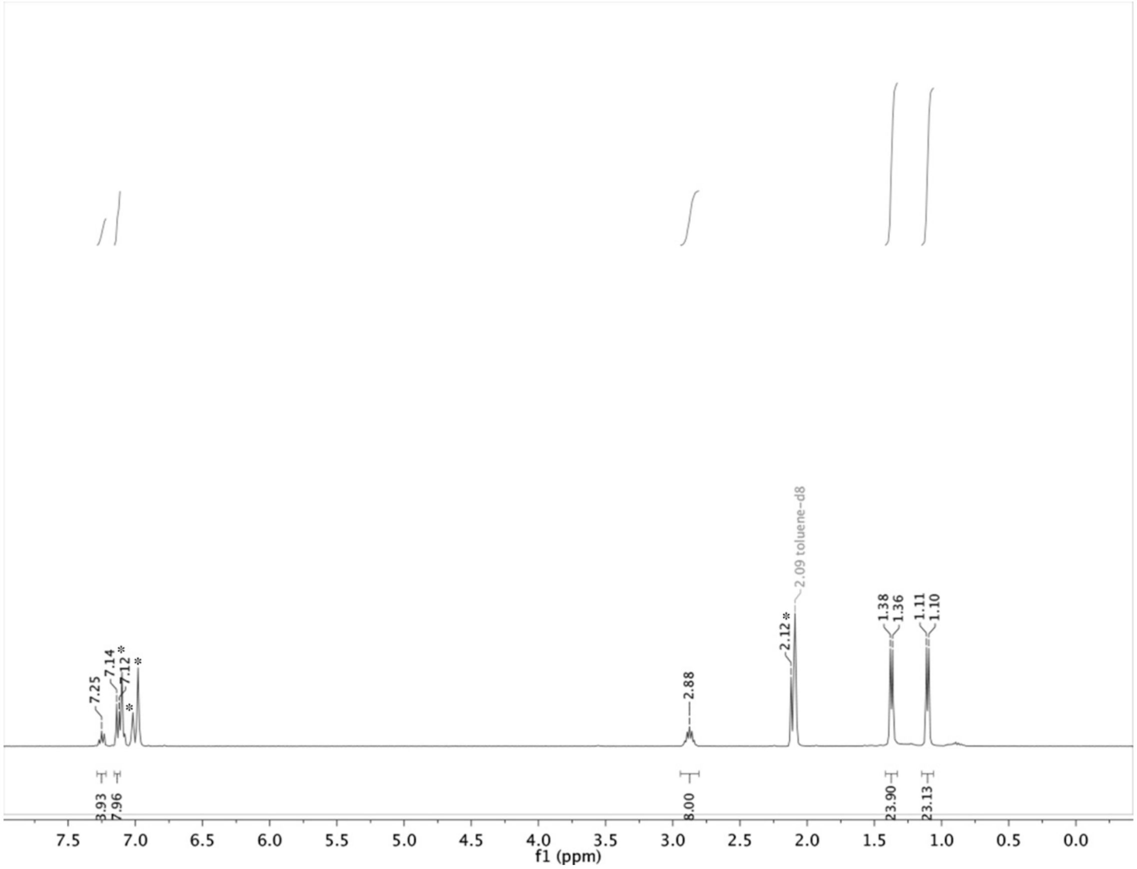

Fig. S10. ^{1}H NMR spectrum of **5** in toluene-d_8 at 25 ºC (*: toluene).

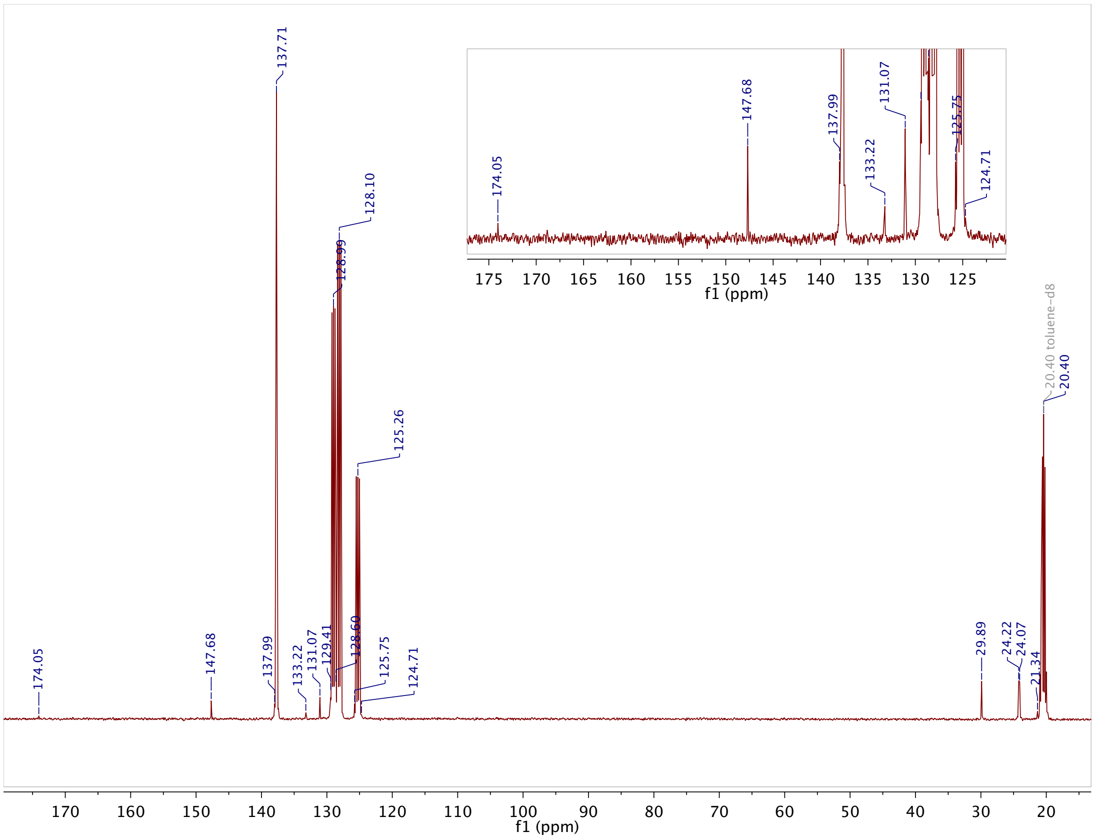

Fig. S11. ^{13}C NMR spectrum of **5** in toluene-d_8 at 100 ºC (resonances of toluene solvent: 21.34, 125.75, 128.60, 129.41, 137.99 ppm).

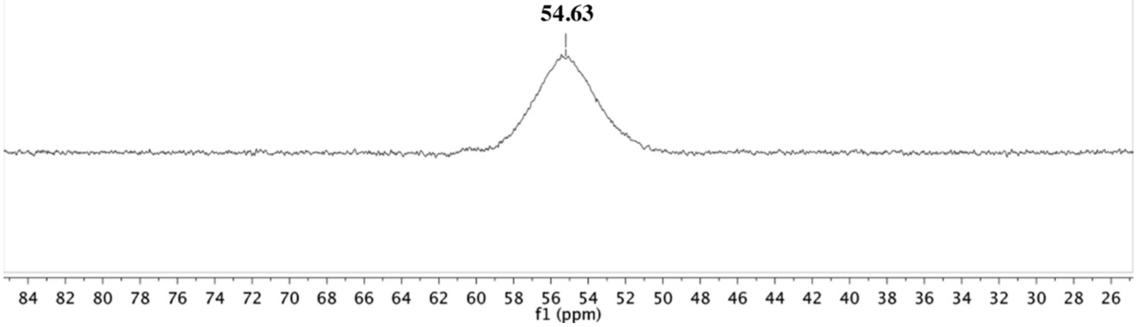

Fig. S12. $^{11}B\{^{1}H\}$ NMR spectrum of **5** in toluene-d$_8$ at 100 ºC.

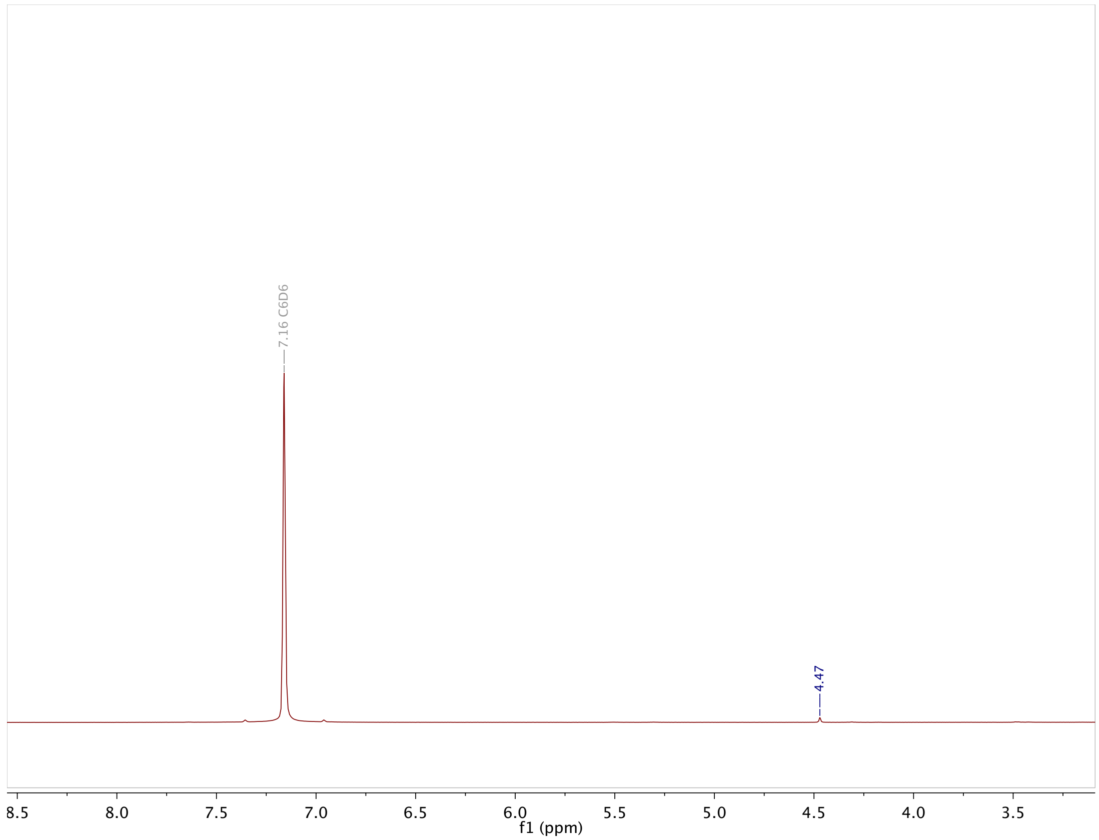

Fig. S13. ^{1}H NMR spectrum of H_2 in C_6D_6 at 25 ºC.

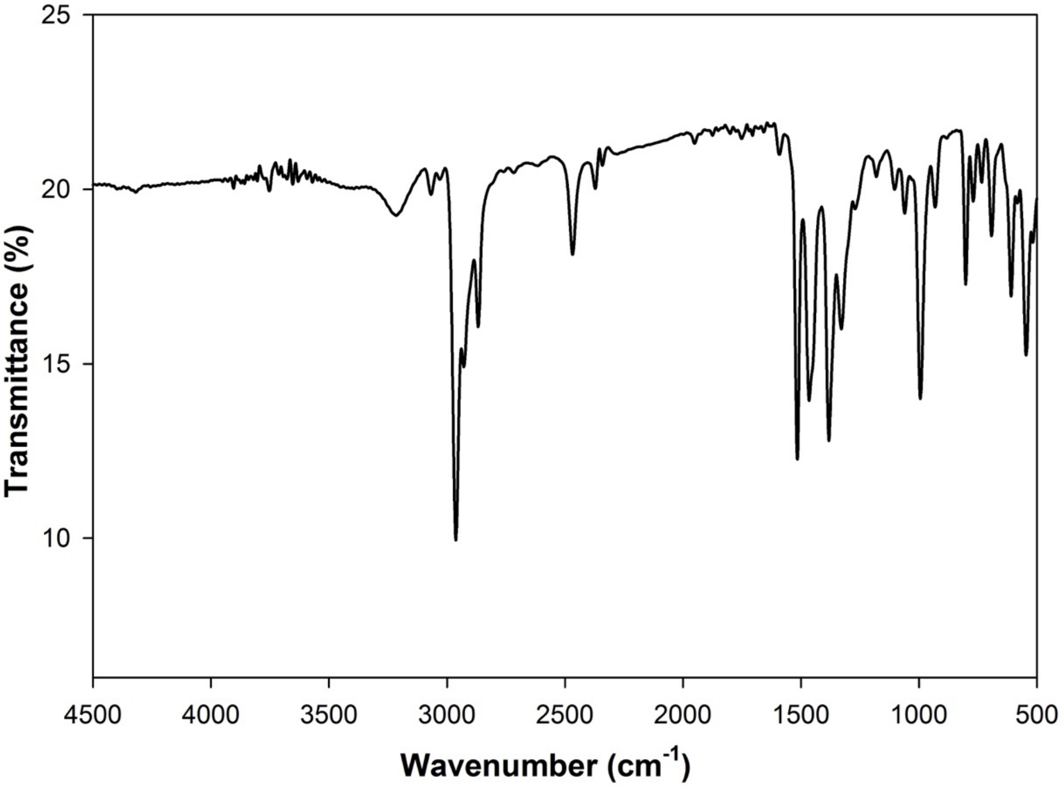

Fig. S14. IR(KBr) spectrum of **2**.

SUPPORTING INFORMATION of COMPUTATIONS

All computations employed the Gaussian 16 (Revision C.01) program:

Gaussian 16, Revision C.01,
M. J. Frisch, G. W. Trucks, H. B. Schlegel, G. E. Scuseria,
M. A. Robb, J. R. Cheeseman, G. Scalmani, V. Barone,
G. A. Petersson, H. Nakatsuji, X. Li, M. Caricato, A. V. Marenich,
J. Bloino, B. G. Janesko, R. Gomperts, B. Mennucci, H. P. Hratchian,
J. V. Ortiz, A. F. Izmaylov, J. L. Sonnenberg, D. Williams-Young,
F. Ding, F. Lipparini, F. Egidi, J. Goings, B. Peng, A. Petrone,
T. Henderson, D. Ranasinghe, V. G. Zakrzewski, J. Gao, N. Rega,
G. Zheng, W. Liang, M. Hada, M. Ehara, K. Toyota, R. Fukuda,
J. Hasegawa, M. Ishida, T. Nakajima, Y. Honda, O. Kitao, H. Nakai,
T. Vreven, K. Throssell, J. A. Montgomery, Jr., J. E. Peralta,
F. Ogliaro, M. J. Bearpark, J. J. Heyd, E. N. Brothers, K. N. Kudin,
V. N. Staroverov, T. A. Keith, R. Kobayashi, J. Normand,
K. Raghavachari, A. P. Rendell, J. C. Burant, S. S. Iyengar,
J. Tomasi, M. Cossi, J. M. Millam, M. Klene, C. Adamo, R. Cammi,
J. W. Ochterski, R. L. Martin, K. Morokuma, O. Farkas,
J. B. Foresman, and D. J. Fox, Gaussian, Inc., Wallingford CT, 2019.

SUPPORTING INFORMATION of X-RAY

Compound **2·(toluene)**

Table S1. Sample and crystal data for **2·(toluene)**.

**Identification code**	**2·(toluene)**	
**Chemical formula**	$C_{34}H_{43}BN_2S_3$	
**Formula weight**	586.69 g/mol	
**Temperature**	299(2) K	
**Wavelength**	0.71073 Å	
**Crystal size**	0.120 x 0.210 x 0.290 mm	
**Crystal system**	monoclinic	
**Space group**	P2/c (No. 13)	
**Unit cell dimensions**	a = 10.7109(8) Å	α = 90°
	b = 10.5096(8) Å	β = 107.465(2)°
	c = 16.2052(12) Å	γ = 90°
**Volume**	1740.1(2) $Å^3$	
**Z**	2	
**Density (calculated)**	1.120 g/cm^3	
**Absorption coefficient**	0.237 mm^{-1}	
**F(000)**	628	

Table S2. Data collection and structure refinement for **2·(toluene)**.

**Theta range for data collection**	1.94 to 28.28°	
**Index ranges**	-14<=h<=14, -14<=k<=14, -21<=l<=21	
**Reflections collected**	48471	
**Independent reflections**	4309 [R(int) = 0.1078]	
**Max. and min. transmission**	0.7458 and 0.4827	
**Structure solution technique**	direct methods	
**Structure solution program**	SHELXT 2014/5 (Sheldrick, 2014)	
**Refinement method**	Full-matrix least-squares on F^2	
**Refinement program**	SHELXL-2018/3 (Sheldrick, 2018)	
**Function minimized**	$\Sigma w(F_o^2 - F_c^2)^2$	
**Data / restraints / parameters**	4309 / 2 / 205	
**Goodness-of-fit on F^2**	1.010	
**Δ/σ_{max}**	0.003	
**Final R indices**	2629 data; $I>2\sigma(I)$	R1 = 0.0676, wR2 = 0.1623
	all data	R1 = 0.1164, wR2 = 0.1973
**Weighting scheme**	$w=1/[\sigma^2(F_o^2)+(0.0830P)^2+1.1650P]$<br>where $P=(F_o^2+2F_c^2)/3$	
**Largest diff. peak and hole**	0.391 and -0.277 eÅ^{-3}	
**R.M.S. deviation from mean**	0.049 eÅ^{-3}	

Table S3. Bond lengths (Å) for **2·(toluene)**.

S1-C1	1.655(4)	S2-C2	1.724(2)
S2-B1	1.792(3)	B1-H1	1.15(2)
N1-C1	1.372(3)	N1-C2	1.384(3)
N1-C3	1.444(3)	C2-C2#1	1.331(5)
C3-C8	1.380(4)	C3-C4	1.386(4)
C4-C5	1.391(4)	C4-C12	1.510(4)
C5-C6	1.358(5)	C6-C7	1.362(5)
C7-C8	1.402(4)	C8-C9	1.494(4)
C9-C10	1.498(6)	C9-C11	1.516(5)
C12-C14	1.509(6)	C12-C13	1.513(6)
C15-C16	1.39	C15-C20	1.39
C15-C21	1.37(3)	C16-C17	1.39
C17-C18	1.39	C18-C19	1.39
C19-C20	1.39		

Symmetry transformations used to generate equivalent atoms:

#1 -x+1, y, -z+3/2

Table S4. Bond angles (°) for **2·(toluene)**.

C2-S2-B1	92.17(16)	H1-B1-S2	121.93(14)
H1-B1-S2#1	121.93(14)	S2-B1-S2#1	116.1(3)
C1-N1-C2	109.1(2)	C1-N1-C3	124.6(2)
C2-N1-C3	126.29(19)	N1#1-C1-N1	105.7(3)
N1#1-C1-S1	127.17(14)	N1-C1-S1	127.17(14)
C2#1-C2-N1	108.03(13)	C2#1-C2-S2	119.74(8)
N1-C2-S2	132.22(18)	C8-C3-C4	124.3(2)
C8-C3-N1	118.0(2)	C4-C3-N1	117.8(2)
C3-C4-C5	116.2(3)	C3-C4-C12	123.2(2)
C5-C4-C12	120.6(3)	C6-C5-C4	121.4(3)
C7-C6-C5	120.9(3)	C6-C7-C8	120.8(3)
C3-C8-C7	116.3(3)	C3-C8-C9	122.7(2)
C7-C8-C9	121.0(3)	C8-C9-C10	112.6(3)
C8-C9-C11	111.7(3)	C10-C9-C11	112.7(4)
C4-C12-C14	111.6(3)	C4-C12-C13	111.6(3)
C14-C12-C13	111.4(4)	C16-C15-C20	120.0
C16-C15-C21	116.3(18)	C20-C15-C21	123.1(18)
C15-C16-C17	120.0	C18-C17-C16	120.0
C19-C18-C17	120.0	C18-C19-C20	120.0
C19-C20-C15	120.0		

Symmetry transformations used to generate equivalent atoms:

#1 -x+1, y, -z+3/2

Compound **4**

Table S5. Sample and crystal data for **4**.

**Identification code**	**4**	
**Chemical formula**	$C_{81}H_{102}B_2N_6S_9$	
**Formula weight**	1469.84 g/mol	
**Temperature**	135(2) K	
**Wavelength**	0.71073 Å	
**Crystal size**	0.150 x 0.220 x 0.300 mm	
**Crystal system**	monoclinic	
**Space group**	C2/c (No. 15)	
**Unit cell dimensions**	a = 12.260(2) Å	α = 90°
	b = 26.520(4) Å	β = 96.180(5)°
	c = 34.227(5) Å	γ = 90°
**Volume**	11063(3) $Å^3$	
**Z**	4	
**Density (calculated)**	0.882 g/cm^3	
**Absorption coefficient**	0.214 mm^{-1}	
**F(000)**	3136	

Table S6. Data collection and structure refinement for **4**.

**Theta range for data collection**	1.84 to 25.50°
**Index ranges**	-14<=h<=14, -32<=k<=32, -41<=l<=40
**Reflections collected**	64555
**Independent reflections**	10265 [R(int) = 0.0741]
**Max. and min. transmission**	0.7456 and 0.4941
**Structure solution technique**	direct methods
**Structure solution program**	SHELXT 2014/5 (Sheldrick, 2014)
**Refinement method**	Full-matrix least-squares on F^2
**Refinement program**	SHELXL-2018/3 (Sheldrick, 2018)
**Function minimized**	$\Sigma w(F_o^2 - F_c^2)^2$
**Data / restraints / parameters**	10265 / 0 / 443
**Goodness-of-fit on F^2**	1.080
**Final R indices**	8559 data; I>2σ(I) R1 = 0.0880, wR2 = 0.2378
	all data R1 = 0.1000, wR2 = 0.2469
**Weighting scheme**	$w=1/[\sigma^2(F_o^2)+(0.0986P)^2+81.9767P]$ where $P=(F_o^2+2F_c^2)/3$
**Largest diff. peak and hole**	0.461 and -0.443 eÅ^{-3}
**R.M.S. deviation from mean**	0.099 eÅ^{-3}

Table S7. Bond lengths (Å) for **4**.

S1-C1	1.658(7)	S2-C2	1.759(4)
S2-B1	1.815(5)	S3-C15	1.659(4)
S4-C16	1.735(4)	S4-B1	1.812(5)
S5-C17	1.733(4)	S5-B1	1.813(4)
N1-C1	1.370(5)	N1-C2	1.386(5)
N1-C3	1.448(5)	N2-C15	1.383(5)
N2-C16	1.393(5)	N2-C30	1.436(5)
N3-C15	1.380(5)	N3-C17	1.384(5)
N3-C18	1.443(5)	C2-C2#1	1.342(8)
C3-C4	1.386(7)	C3-C8	1.408(6)
C4-C5	1.385(7)	C4-C12	1.528(6)
C5-C6	1.390(7)	C6-C7	1.365(7)
C7-C8	1.417(6)	C8-C9	1.515(7)
C9-C10	1.523(7)	C9-C11	1.526(7)
C12-C13	1.521(7)	C12-C14	1.544(7)
C16-C17	1.341(6)	C18-C19	1.384(6)
C18-C23	1.401(6)	C19-C20	1.407(6)
C19-C27	1.529(7)	C20-C21	1.381(8)
C21-C22	1.385(8)	C22-C23	1.398(7)
C23-C24	1.515(7)	C24-C26	1.532(8)
C24-C25	1.531(8)	C27-C29	1.508(8)
C27-C28	1.540(7)	C30-C31	1.397(7)
C30-C35	1.398(7)	C31-C32	1.391(7)
C31-C39	1.519(7)	C32-C33	1.381(9)
C33-C34	1.387(10)	C34-C35	1.400(8)
C35-C36	1.519(8)	C36-C37	1.539(8)
C36-C38	1.524(8)	C39-C41	1.525(7)
C39-C40	1.534(8)		

Symmetry transformations used to generate equivalent atoms:
#1 -x+1, y, -z+1/2

Table S8. Bond angles (°) for **4**.

C2-S2-B1	100.4(2)	C16-S4-B1	91.3(2)
C17-S5-B1	92.0(2)	S5-B1-S4	116.4(2)
S5-B1-S2	123.9(3)	S4-B1-S2	119.7(2)
C1-N1-C2	109.6(4)	C1-N1-C3	123.5(4)
C2-N1-C3	126.8(3)	C15-N2-C16	108.5(3)
C15-N2-C30	126.7(3)	C16-N2-C30	124.8(3)
C15-N3-C17	109.7(3)	C15-N3-C18	124.9(3)
C17-N3-C18	125.3(4)	N1#1-C1-N1	105.5(5)
N1#1-C1-S1	127.3(3)	N1-C1-S1	127.3(3)
C2#1-C2-N1	107.6(2)	C2#1-C2-S2	130.58(14)
N1-C2-S2	121.8(3)	C4-C3-C8	122.8(4)
C4-C3-N1	120.0(4)	C8-C3-N1	117.2(4)
C5-C4-C3	118.3(4)	C5-C4-C12	120.4(4)
C3-C4-C12	121.3(4)	C4-C5-C6	120.8(5)
C7-C6-C5	120.3(4)	C6-C7-C8	121.4(4)
C3-C8-C7	116.3(4)	C3-C8-C9	123.0(4)
C7-C8-C9	120.7(4)	C10-C9-C8	112.2(4)
C10-C9-C11	110.5(5)	C8-C9-C11	111.2(4)
C4-C12-C13	110.2(4)	C4-C12-C14	110.6(4)
C13-C12-C14	111.0(5)	N2-C15-N3	105.7(3)
N2-C15-S3	127.1(3)	N3-C15-S3	127.2(3)
C17-C16-N2	108.6(3)	C17-C16-S4	120.9(3)
N2-C16-S4	130.5(3)	C16-C17-N3	107.5(4)
C16-C17-S5	119.3(3)	N3-C17-S5	133.2(3)
C19-C18-C23	123.9(4)	C19-C18-N3	118.8(4)
C23-C18-N3	117.3(4)	C18-C19-C20	117.7(4)
C18-C19-C27	123.1(4)	C20-C19-C27	119.2(4)
C21-C20-C19	119.8(5)	C22-C21-C20	121.3(5)
C21-C22-C23	120.8(5)	C22-C23-C18	116.6(5)
C22-C23-C24	120.3(4)	C18-C23-C24	123.1(4)
C23-C24-C26	110.7(4)	C23-C24-C25	111.7(5)
C26-C24-C25	111.1(5)	C29-C27-C19	111.5(4)
C29-C27-C28	110.9(5)	C19-C27-C28	110.4(4)
C31-C30-C35	124.5(4)	C31-C30-N2	117.2(4)
C35-C30-N2	118.1(4)	C30-C31-C32	116.7(5)
C30-C31-C39	121.4(4)	C32-C31-C39	121.8(5)
C31-C32-C33	120.9(6)	C34-C33-C32	120.7(5)
C35-C34-C33	121.2(6)	C34-C35-C30	115.9(5)
C34-C35-C36	123.3(5)	C30-C35-C36	120.8(4)
C35-C36-C37	111.1(4)	C35-C36-C38	113.3(5)
C37-C36-C38	109.1(5)	C31-C39-C41	114.1(4)
C31-C39-C40	111.3(5)	C41-C39-C40	110.0(5)

Symmetry transformations used to generate equivalent atoms:
#1 -x+1, y, -z+1/2

Compound **5·(toluene)$_2$**

Table S9. Sample and crystal data for **5·(toluene)$_2$**.

**Identification code**	**5·(toluene)$_2$**	
**Chemical formula**	$C_{68}H_{84}B_2N_4S_6$	
**Formula weight**	1171.36 g/mol	
**Temperature**	299(2) K	
**Wavelength**	0.71073 Å	
**Crystal size**	0.140 x 0.230 x 0.270 mm	
**Crystal system**	triclinic	
**Space group**	P-1 (No. 2)	
**Unit cell dimensions**	a = 9.0772(15) Å	α = 112.525(4)°
	b = 13.779(2) Å	β = 96.635(5)°
	c = 14.937(3) Å	γ = 92.610(4)°
**Volume**	1705.9(5) Å^3	
**Z**	1	
**Density (calculated)**	1.140 g/cm^3	
**Absorption coefficient**	0.241 mm^{-1}	
**F(000)**	626	

Table S10. Data collection and structure refinement for **5·(toluene)$_2$**.

**Theta range for data collection**	2.27 to 27.88°	
**Index ranges**	-11<=h<=11, -18<=k<=18, -19<=l<=19	
**Reflections collected**	52236	
**Independent reflections**	8121 [R(int) = 0.0980]	
**Max. and min. transmission**	0.7457 and 0.5649	
**Structure solution technique**	direct methods	
**Structure solution program**	SHELXT 2014/5 (Sheldrick, 2014)	
**Refinement method**	Full-matrix least-squares on F^2	
**Refinement program**	SHELXL-2018/3 (Sheldrick, 2018)	
**Function minimized**	$\Sigma w(F_o^2 - F_c^2)^2$	
**Data / restraints / parameters**	8121 / 1 / 350	
**Goodness-of-fit on F^2**	1.025	
**Δ/σ_{max}**	0.003	
**Final R indices**	4961 data; I>2σ(I)	R1 = 0.0700, wR2 = 0.1743
	all data	R1 = 0.1230, wR2 = 0.2090
**Weighting scheme**	$w=1/[\sigma^2(F_o^2)+(0.0970P)^2+0.9645P]$<br>where $P=(F_o^2+2F_c^2)/3$	
**Largest diff. peak and hole**	0.537 and -0.392 eÅ^{-3}	
**R.M.S. deviation from mean**	0.060 eÅ^{-3}	

Table S11. Bond lengths (Å) for **5·(toluene)₂**.

S1-C1	1.652(3)	S2-C2	1.715(3)
S2-B1	1.796(3)	S3-C3	1.716(3)
S3-B1	1.801(3)	B1-B1#1	1.645(6)
N1-C2	1.376(3)	N1-C1	1.371(3)
N1-C16	1.444(3)	N2-C3	1.375(3)
N2-C1	1.374(3)	N2-C4	1.444(4)
C2-C3	1.333(4)	C4-C5	1.389(5)
C4-C9	1.376(5)	C5-C6	1.387(5)
C5-C13	1.498(6)	C6-C7	1.348(8)
C7-C8	1.342(9)	C8-C9	1.404(6)
C9-C10	1.502(7)	C10-C12	1.536(7)
C10-C11	1.509(7)	C13-C14	1.490(7)
C13-C15	1.508(7)	C16-C17	1.381(4)
C16-C21	1.387(4)	C17-C18	1.384(5)
C17-C25	1.514(5)	C18-C19	1.367(6)
C19-C20	1.358(6)	C20-C21	1.384(5)
C21-C22	1.498(5)	C22-C24	1.511(8)
C22-C23	1.509(7)	C25-C27	1.518(6)
C25-C26	1.518(6)	C28-C29	1.39
C28-C33	1.39	C28-C34	1.437(8)
C29-C30	1.39	C30-C31	1.39
C31-C32	1.39	C32-C33	1.39

Symmetry transformations used to generate equivalent atoms:
#1 -x+2, -y+1, -z+2

Table S12. Bond angles (°) for **5·(toluene)$_2$**.

C2-S2-B1	93.82(14)	C3-S3-B1	93.59(14)
B1#1-B1-S3	122.5(3)	B1#1-B1-S2	123.6(3)
S3-B1-S2	113.88(16)	C2-N1-C1	109.3(2)
C2-N1-C16	125.0(2)	C1-N1-C16	125.7(2)
C3-N2-C1	109.2(2)	C3-N2-C4	125.8(2)
C1-N2-C4	125.0(2)	N2-C1-N1	105.4(2)
N2-C1-S1	127.3(2)	N1-C1-S1	127.4(2)
C3-C2-N1	108.0(2)	C3-C2-S2	119.2(2)
N1-C2-S2	132.7(2)	C2-C3-N2	108.0(2)
C2-C3-S3	119.5(2)	N2-C3-S3	132.5(2)
C5-C4-C9	124.7(3)	C5-C4-N2	117.6(3)
C9-C4-N2	117.7(3)	C4-C5-C6	115.3(4)
C4-C5-C13	122.8(3)	C6-C5-C13	121.9(4)
C7-C6-C5	122.4(5)	C6-C7-C8	120.2(4)
C7-C8-C9	122.1(5)	C4-C9-C8	115.2(5)
C4-C9-C10	122.8(3)	C8-C9-C10	122.0(4)
C9-C10-C12	112.4(5)	C9-C10-C11	112.0(4)
C12-C10-C11	110.6(5)	C14-C13-C5	113.8(4)
C14-C13-C15	110.8(5)	C5-C13-C15	111.4(5)
C17-C16-C21	124.1(3)	C17-C16-N1	118.2(3)
C21-C16-N1	117.7(3)	C18-C17-C16	116.3(3)
C18-C17-C25	121.5(3)	C16-C17-C25	122.2(3)
C17-C18-C19	121.1(4)	C20-C19-C18	121.1(3)
C19-C20-C21	120.8(4)	C16-C21-C20	116.6(3)
C16-C21-C22	122.5(3)	C20-C21-C22	120.9(3)
C21-C22-C24	110.8(4)	C21-C22-C23	113.6(4)
C24-C22-C23	110.9(4)	C17-C25-C27	113.1(4)
C17-C25-C26	110.3(3)	C27-C25-C26	111.6(4)
C29-C28-C33	120.0	C29-C28-C34	122.4(8)
C33-C28-C34	117.6(8)	C28-C29-C30	120.0
C31-C30-C29	120.0	C30-C31-C32	120.0
C31-C32-C33	120.0	C32-C33-C28	120.0

Symmetry transformations used to generate equivalent atoms:
#1 -x+2, -y+1, -z+2

References:

1. Gruden, E.; Tavcar, G., Synthesis and Characterization of Partially Substituted NHC Supported Alane Adducts Using Triflate or Chloride Salts. *Polyhedron* **2021,** *196*, 115009.
